# Telemedicine in war zones: prospects, barriers, and meeting the needs of special populations

**DOI:** 10.3389/fmed.2024.1417025

**Published:** 2024-10-10

**Authors:** Motti Haimi

**Affiliations:** ^1^Health Systems Management Department, The Max Stern Yezreel Valley College, Emek Yezreel, Israel; ^2^Rappaport Faculty of Medicine, Technion-Israel Institute of Technology, Haifa, Israel; ^3^ISfTeH - International Society for Telemedicine and eHealth, Basel, Switzerland; ^4^Meuhedet Health Services, Haifa, Israel

**Keywords:** telemedicine, war zones, elderly, special population groups, routine, prospects

## Introduction

Telemedicine is the application of electronic communications to transfer medical information across locations in order to enhance a patient's medical condition (1).

Since its development in the 1950s, the industry has expanded to become a diverse range of services utilized in private homes, medical offices, and hospitals worldwide. Beyond the conventional requirement for remote care, its application has grown to address the difficulty in accessing healthcare specialists (1).

The use of telemedicine has numerous advantages. Access to specialized care could be made easier with the use of telemedicine. Not only does telemedicine facilitate easy access to medical professionals, but it also permits primary care physicians to confer with specialists and smaller hospitals to contract out the evaluation of x-rays to specialists. In the end, telemedicine will increase specialized care accessibility and cultivate a more intimate doctor-patient bond. Patients will be given the confidence to oversee their own care and take control of their health (2–5).

Globally, telemedicine consultations have become increasingly popular among medical professionals as a result of the coronavirus pandemic (COVID-19). The use of special applications like WhatsApp to consult with specialists has become more popular than ever since social distancing has become a significant technique for reducing the danger of COVID-19 exposure for patients and providers (6–8).

However, telemedicine still has disadvantages despite its possible benefits. Among the possible drawbacks are poor coverage, greater initial expenses, and the inability to modify workflows. Furthermore, not all patients have access to it, and a large number of them are underprivileged or lack computer literacy (9).

Telemedicine, however, has a promising future because to new technologies. This trend can only get stronger over time, and if decision-makers are determined to see it through to implementation, it will expand and be extensively adopted (9).

In challenging and dangerous circumstances, telemedicine is an invaluable tool that can help save lives. For example, in a number of contexts, such as conflict zones and during natural catastrophes, telemedicine is a worthful instrument.

Because it makes it possible for persons who are in danger to receive medical attention, telemedicine is particularly helpful in these situations. Telemedicine can be utilized in conflict zones to treat soldiers who are sick or injured medically as well as to support soldiers psychologically as they cope with the stress of fighting.

Telemedicine can be utilized in a natural disaster to treat individuals who are trapped or have trouble receiving medical care. Support for mental health can also be given via telemedicine to those who are struggling with the stress of a natural catastrophe.

Telemedicine is frequently utilized in conflict areas to help soldiers with their mental health: for soldiers, talking to someone face-to-face about their experiences and emotions might be challenging. However, they can consult with a counselor via telemedicine while remaining secure within their own base (10).

However, while telemedicine offers a viable remedy for many of the problems in the complicated global healthcare system, it also creates new ones. In the case of elderly patients who are not accustomed to using technology, telemedicine presents an additional technological challenge to the already complicated field of healthcare (2, 9).

## Opportunities and challenges in telemedicine usage in routine times

The last 10 years have seen significant advancements in technology that have fundamentally altered the way we communicate, work, and live. Over 90% of people in developed nations have a mobile phone, and over 65% of Americans own a smartphone. These percentages are even greater among healthcare professionals. Recent years have seen telemedicine become a crucial component in the delivery of healthcare (11, 12).

Prior to these technological advancements, a patient had to schedule an in-person visit in order to consult a medical specialist. The result of this was decreased access to healthcare professionals, extended wait periods, missed workdays and unsatisfactory patient care. Healthcare practitioners have been looking for alternatives to this time-consuming procedure lately, particularly through telemedicine. These days, social media or instant messaging apps allow patients and Primary Care Physicians (PCPs) to communicate directly with medical specialists, which may shorten the time it takes for a diagnosis and subsequent therapy (13–15).

The World Health Organization (WHO) has long supported telemedicine, and in 2005 it established the Global Observatory for eHealth, which includes telemedicine. The Sustainable Development Goals on Universal Health Coverage are one of the objectives for which WHO has promoted the use of eHealth solutions (the use of information and communication technologies for health). Telecardiology, teleradiology, tele-mental health, tele-intensive care unit (ICU), and teledermatology are a few instances of telemedicine interventions in low- and middle-income countries (LMICs) (16–18).

Although telemedicine may not be suitable for all patients, research indicates that a majority of doctors and patients endorse it (19, 20).

Although a study of clinical trials employing video teleconferencing found broadly identical outcomes, researchers are still looking into whether patients using the virtual services fare as well as those receiving in-person care (21).

Video consultations, phone conversations, secure texting, and wearable-based remote patient monitoring are examples of traditional telehealth services. In addition to the clinician-to-patient category of telehealth services, Healthcare & Public Health Sector Coordinating Councils emphasize the significance of clinician-to-clinician services, which involve clinicians communicating with one another to exchange clinical information and talk about patient care, telementoring, and training (22, 23).

During COVID-19, telehealth proved to be advantageous for both the general public and senior citizens in the United States. In a qualitative review, doctors noted that telemedicine was quickly and iteratively adopted, with utilization waning as the prevalence of coronavirus sickness in 2019 decreased. Interventions and regulations that support telehealth for continuous healthcare delivery and guarantee its accessibility for senior citizens can be informed by physician experiences during the epidemic (20).

Although telemedicine presents a promising solution to numerous issues in the worldwide malfunctioning healthcare system, it also introduces new ones. Telemedicine adds a technological hurdle to the already complex field of health care, particularly for older people who are not used to using technology (24, 25).

The integration of telemedicine solutions in low- and middle-income countries (LMICs) or conflict areas is hindered by various factors such as inadequate financial, material, and human resources, unstable electrical supply, and poor connection. Additional issues include inadequate implementation, a lack of effect evaluation, and a weak body of data, especially in contexts influenced by conflict (18).

In order to guarantee fair access to health care, especially during pandemics like the COVID-19, the main obstacles to the use of telemedicine, such as lower educational levels, digital literacy skills, or access to internet connectivity, must be overcome (26–28).

Even if more sophisticated technology is available, it is crucial to offer low-tech, user-friendly solutions for individuals who are less tech-savvy in order to increase acceptance even further (24, 25). Accordingly, the experience in Ukraine demonstrates that, out of the 62,000 telemedicine consultations carried out by *Telehelp Ukraine* and *Health Tech Without Borders*, 98% of them were limited to text- based contact between the patient and the physician via a secure telehealth platform (29).

There are also barriers to telemedicine adoption by the physicians themselves, especially when it perceived as high-risk activity involving difficulties on making proper diagnosis (30, 31).

In addition to the challenges associated with remote diagnosis, there are circumstances in which remote counseling is inappropriate, and there are worries regarding the abuse or misuse of this platform, which could hinder patients' access to in-person medical surveillance in the clinic and jeopardize their follow-up and medical interventions, which are not always feasible to monitor remotely. Furthermore, physicians have reported that certain objective measures of care quality, such as blood pressure, glucose, or cholesterol measurements, may decline in quality and frequency as they have to rely on patients to report and maintain their vitals through home monitoring devices during telehealth visits (32).

## Telemedicine in war zones–Opportunities and advantages

Telemedicine has emerged as a vital tool in war zones, enabling healthcare professionals to provide care remotely and bridge the gap created by conflict-induced disruptions to traditional healthcare systems. The use of telehealth technologies, such as video conferencing, remote patient monitoring, and teleconsultations, has proven invaluable in delivering essential medical services in situations where access to physical facilities and medical personnel is limited.

During armed conflicts, telemedicine can play a crucial role in providing primary care and managing chronic conditions, facilitating mental health support, offering specialized consultations for complex cases and even training and supporting local healthcare providers (33–36).

Telemedicine can be useful in providing access to persons affected by conflict or in helping those affected by the conflict to access medical experts in dangerous and difficult-to-reach places. This is crucial for older adults who struggle physically to access the medical care they require, both in normal circumstances and during times of conflict. For this population, telemedicine can also provide mental support, which is very important in these situations (33–38).

Getting healthcare professionals to enter or remain in a war zone is extremely difficult because they are already a scarce resource. Therefore, individuals who stayed behind can receive healthcare thanks to telemedicine. The majority of individuals in conflict areas use their phones, and all they need for a telemedicine call is phone connectivity and cellular access (33–36).

Long-lasting, intricate armed conflicts have a negative impact on the health of the populace as well as local health systems; these impacts include harm to the infrastructure of healthcare facilities and the departure or death of healthcare professionals, creating a labor shortage. The health of the populace is progressively harmed by this, especially in cases where the most skilled or specialized healthcare professionals have been forcibly relocated, which has an impact on the education and training of less experienced healthcare professionals. When specialized input is needed or there is a shortage of healthcare staff, telemedicine might offer the possibility of remote support. In conflict-affected areas, it can offer a creative, affordable, and reliable form of help; yet, its implementation may be hampered by a lack of local human resources, infrastructure, and connectivity (10, 33–36).

In Ukraine, numerous hospitals suffered damage, and emergency medical personnel were attacked. Internally displaced people make up about 6.5 million of the citizenries. The majority of them live in the areas that are experiencing hostilities and the occupied territory. The most pertinent method of getting health care at this time is remote, given the conditions. Telemedicine's ability to provide access to medical care under such extreme situations is what allows for a significant impact on the situation. This judgment is made naturally by Ukrainian doctors. Typically, they use messengers and phones to offer their patients the ability to independently seek guidance. On social media, doctors have made hundreds of these kinds of offers. Medical specialists and family physicians are among them (29).

Numerous healthcare organizations have launched programs that allow physicians to consult remotely. During peacetime, the majority of them also made active use of these consultation tools. Using telemedicine at a time of war, they were able to expeditiously optimize the treatment and diagnostic process thanks to this expertise.

The experience in Ukraine shows that, of the 62,000 telemedicine consultations conducted by *Health Tech Without Borders* (HTWB) and *Telehelp* Ukraine, 98% of them consisted solely of text communication between the doctor and patient on a secure telehealth platform (29).

Telemedicine solutions also offer psychological support to the populace and avert potential effects, such as post-traumatic stress disorder (22, 29).

Recently, the Ministry of Health of Ukraine launched an additional initiative whereby anyone can dial a single number and be connected to the appropriate professional.

The continuing Russian-Ukrainian conflict has prompted Ukrainian doctors to employ telemedicine more frequently in order to deliver high-quality care to patients even on the front lines. Physicians in Ukraine employed a range of telemedicine options, with direct phone calls and messengers making up the majority of the workflow. Compared to their less experienced counterparts, experienced medical doctors used gadgets and devices for remote patient monitoring, SMS, and email more frequently (29).

## Telemedicine in war zones—Barriers and challenges

Despite the many advantages of using telemedicine in war zones, it is important to note that the effectiveness of telemedicine in war zones depends on several factors, including the availability of reliable communication infrastructure, the accessibility of appropriate medical equipment, and the ability to overcome language and cultural barriers (10).

### Unique challenges faced by special populations

In the context of armed conflict, certain populations face heightened vulnerabilities and require tailored approaches to healthcare delivery. These special populations include refugees and internally displaced persons (IDPs), children and pregnant women, people with disabilities, elderly individuals, and individuals with chronic illnesses. These groups often lack access to basic healthcare services and may experience compounded health risks due to displacement, trauma, malnutrition, and limited access to essential medications. Addressing their specific needs is paramount in ensuring equitable and effective healthcare delivery in war zones (37).

In emergency situations, these populations can be even more affected: some elderly people are evicted from their homes and disconnected from their natural environment, and from access to their permanent medical system. Therefore, the difficulties in accessibility and availability of remote medical services can worsen existing gaps in routine situations. It is less commonly known that elderly people are more susceptible during humanitarian crises (38).

### Accessibility and connectivity issues

One of the most significant challenges in implementing telemedicine in war zones is the lack of accessibility and connectivity. Conflict often disrupts communication infrastructure, leading to limited or unreliable internet access, particularly in remote or conflict-affected areas. This can hinder the use of telehealth technologies and limit the ability of healthcare providers to connect with patients. To overcome these limitations, innovative solutions are needed, such as utilizing satellite communication systems, deploying mobile clinics equipped with telemedicine capabilities, and developing offline data storage and transmission methods. Investing in robust and resilient communication infrastructure is crucial to ensure the widespread adoption and success of telemedicine in war zones (39, 40).

### Addressing language and cultural barriers

In war zones, diverse populations often reside, creating a complex linguistic and cultural landscape. Telemedicine services must be tailored to meet the specific needs of each population, considering language barriers, cultural sensitivities, and differing healthcare practices. This requires providing language interpretation services, utilizing culturally appropriate materials and communication strategies, recruiting healthcare providers who are fluent in the local languages. Understanding and respecting cultural differences is vital to establishing trust and ensuring effective communication between healthcare providers and patients (10, 41, 42).

In addition, some people, particularly those in the elderly age group, prefer direct communication over indirect communication since they don't trust technology or remote diagnosis, as stated by Haimi et al. (24).

### Ensuring patient privacy and data security

Protecting patient privacy and data security is of paramount importance in telemedicine, especially in conflict zones where information systems may be vulnerable to breaches. The sensitive nature of medical data requires robust security measures to prevent unauthorized access, data leaks, and potential misuse.

This can be achieved through implementing strong encryption protocols for all data transmission, utilizing secure cloud storage solutions, and establishing clear guidelines for data access and sharing. It is also crucial to ensure that patient data is stored and managed in accordance with relevant privacy regulations and ethical principles. This requires close collaboration between healthcare providers, technology developers, and regulatory bodies to ensure responsible and ethical use of telemedicine in war zones (37, 43).

## Telemedicine utilization during peace and war times, considering special populations—The example of Israel and the Palestinian authority

Israel ranks among the top 10 countries in the world for smartphone ownership and internet usage, according to recent surveys, with 76% of smartphone owners indicating frequent use of social media apps (44).

Israel has prioritized telemedicine as a national priority, recognizing its immense potential for a long time. It has done this by creating relevant regulations, allocating substantial resources, and encouraging collaborations between independent researchers, start-up companies, health organizations, and research institutions. Israeli telehealth services are provided by all four HMOs (45).

Additionally, the Israeli army made use of telehealth services: With over 2.5 million military doctor visits expected in 2021, the military will have a difficult time seeing all of the patients dispersed among hundreds of bases across the nation, particularly in the West Bank and the Arava region in southern Israel, where thousands of troops are stationed. In the past year, telemedicine-focused remote medical specialist complexes for soldiers have been established. The complexes have two concurrently operating telemedicine “meeting rooms” and are housed in trailers equipped with high-speed Internet. The medical facilities give the soldiers a private space to consult with doctors, saving them the time it would otherwise need to take time off of duty (46).

Since suicides have been the most common cause of death among military personnel in recent years, the Israel Defense Forces (IDF) is working to enhance the standard of treatment given to soldiers seeking mental health services and is heavily utilizing telepsychiatry (46).

The most recent Israeli war with Hamas began on October 7, when Israeli communities in the south of the country where attacked, resulting in over 1,400 people murdered and 240 hostages. Since the beginning of the war, there is undoubtedly a greater prevalence of mental and behavioral health counseling, as well as PTSD counseling, in Israel. Additionally, there are geographical restrictions that limit access and mobility, which likely contributes to the rise in the use of telemedicine for safety.

In Israel—during the peak of the current war—there were over 100,000 evacuees throughout the country. Some of them made it possible to maintain contact with the family doctor and clinic staff with the help of telemedicine. In such a period of uncertainty, instability and change in the natural environment, it is important to be treated by the regular attending physician, who knows the patient and his family, even remotely (47).

There are special populations (such as the elderly, the ultra-Orthodox, the Arab population)—who, even in routine situations, do not have equal and appropriate access to Internet services, and therefore also to telemedicine services. They also have other difficulties that interfere with the use of telemedicine—literacy, technological and physical difficulties. In Israel, the ability to keep these individuals safe has once again been put to the test during the recent war, in which many of the people injured or displaced from their homes were elderly (48).

Older adults confront numerous additional challenges on top of the basic risks. Even younger, more fitter people may find it difficult to have the speed and agility needed only to get into a bomb shelter. Many elderly people who were injured during these attacks were only heading to a shelter; they suffered fractures to their wrists, hips, and heads. Not to mention feeble people confined to wheelchairs or beds who may only hope that their home will escape damage. Additionally, people suffering from dementia will find it difficult to comprehend what is happening and what to do precisely when the sirens sound (48).

In addition, threats to Israel's southern and northern borders have already resulted in the displacement of 240,000 Israelis of all ages, who are taking refuge in strange places, mostly in hotel settings, occasionally accommodating entire families in a single room. Equipment, glasses, canes, hearing aids, and most importantly, medications, have been left behind in the haste to leave their homes. Furthermore, it is heartbreaking to consider the over thirty elderly people, several more than 80, taken hostage by Hamas and imprisoned in Gaza's underground tunnels (48).

Paradoxically, the latest COVID-19 “adventure” has accelerated advancements in the programs and structures that the current medical system requires, such as telemedicine and improved home care. Moreover, services for senior citizens are well-designed and organized. The Israeli society has evolved unique solutions. For instance, the nation has created a top-notch, highly “wired” health care system with unique services for its senior population. Elderly people who have been displaced can therefore access their medical records through their HMO from any location. However, most people lose touch with their own family doctor, which is crucial for elderly patients with complicated medical histories. Here, the majority of elderly people have strong assistance from their families and neighbors (if not displaced). Particularly during a crisis like this one, social solidarity is high (49).

In the Palestinian Authority, telemedicine has become necessary due to the Palestinian Authority's lack of qualified doctors and healthcare providers. Implementing such networks is crucial due to the difficulty and expense of transport between Palestinian cities (50).

Zatari et al. (50) described sending radiographs from one computer to another during testing. They planned to establish a communication link between Palestinian hospitals at various places.

The advantages of telemedicine are widely recognized in these regions, mostly due to its ability to provide patients in rural or underserved areas with a professional response to their illnesses in a matter of minutes, at most, several hours. Because there are no time zone restrictions for this type of consultation, developing nations will benefit from this technology as they will be able to contact experts worldwide and give their patients better care as a result.

In addition to creating an integrated telecommunications infrastructure, the Palestinian National Strategic Health Plan (PNSHP 1999–2003) has prioritized the development of advanced medical informatics applications, such as telemedicine and electronic medical records (51). Although telemedicine has not yet been created, efforts to promote telemedicine in Palestinian Authority are planned to be advanced through the Global University System. The establishment of telemedicine within the Palestinian- Authority health management information system aims to both lessen the financial burden of treatment abroad, which accounted for 13% of MOH expenditures, and provide medical professionals with the chance to exchange information with other professionals, both locally and internationally (52).

Even though telemedicine has shown to be an effective technique in conflict areas, there are still issues that need to be resolved, especially in these areas. These include overcoming technological obstacles, educating medical staff on telemedicine procedures, and protecting patient privacy and data security.

The Palestine Children's Relief Fund (PCRF) aims to provide humanitarian aid and medical relief to children and their families, some of whom are refugees fleeing their home countries, using many projects (including the use of telemedicine). These efforts help to ensure that children in need get the vital assistance they require (53).

In recent years, there have been multiple telemedicine collaborations between Israeli hospitals and Palestinian patients.

For example, Samson Assuta Ashdod University Hospital opened a clinic, using advanced telemedicine technologies to treat Palestinians over the internet. It is also bringing patients from the West Bank and Gaza into the hospital. According to Prof. Adi Leiba, head of Assuta's Nephrology and Hypertension Institute and deputy director of the hospital: “No matter what the political situation or how much tension, we are persistent in the fact that we want to bring health to everyone” (54).

Another example is the collaboration between OB-GYN Beyond, Sheba's virtual OBGYN department and the co-existence non-profit Project *Rozana*, which set up a remote OB-GYN unit in the rural Hebron area and are fully operated by Palestinian healthcare teams (55).

The Middle East Ophthalmology Network, which operates in ten significant ophthalmology centers across Israel, Jordan, Morocco, the Palestinian Authority, and Tunisia, is another illustration. About fifty doctors from the Middle East were given the chance to collaborate on clinical consultations for diagnosis and treatment decisions across geographical and political barriers as a result of the project (56).

These instances demonstrate how telemedicine can help to overcome divides in geography, politics, and culture about the common goal of achieving the best possible health results.

## Discussion

Telemedicine has many benefits, in routine times and in emergency times like pandemics and war.

In the case of elderly patients who are not accustomed to using technology, telemedicine presents an additional technological challenge to the already complicated field of healthcare.

These populations may be much more impacted in an emergency: some elderly people may face homelessness, be cut off from their natural surroundings, and lose access to their long-term healthcare system. As a result, the challenges associated with providing remote medical treatments may make already-existing gaps in everyday circumstances worse.

The main concern for legislators will not be whether or not to permit telehealth, but rather how to make it universally accessible, efficient, and egalitarian. Legislators need to be aware of the obstacles and issues that telemedicine presents, particularly when it comes to vulnerable populations like the elderly. Localized interventions that are suitable for the environment and the needs of the local people and health professionals are clearly needed.

It is imperative for policymakers to furnish these populations with the requisite resources so they can obtain services that are accessible, egalitarian, and available.

Another important consideration is integrating telemedicine with existing healthcare systems. This is essential to create a comprehensive and sustainable approach to healthcare delivery in war zones. This integration should involve a seamless flow of information, coordinated care plans, and efficient referral mechanisms. It is also important to ensure that telemedicine services complement, rather than replace, traditional healthcare approaches.

Examples of effective integration include using telemedicine to conduct initial assessments and triage patients, facilitating remote consultations with specialists, and providing ongoing monitoring and follow-up care for patients with chronic conditions. By integrating telemedicine seamlessly with traditional healthcare systems, we can maximize its impact and create a more resilient and responsive healthcare infrastructure in war zones.

Effective implementation of telemedicine requires adequately trained and equipped healthcare providers. This involves providing training on the use of telemedicine technologies, as well as the ethical and legal considerations associated with providing care remotely. It is also important to offer ongoing support to providers to address technical issues, ensure adherence to protocols, and facilitate collaboration with specialists.

Training programs should be tailored to the specific needs and contexts of war zones, considering factors such as the availability of reliable internet access, the language skills of healthcare providers, and the cultural sensitivities of the population. Adequate training and support are crucial in fostering confidence and competence among healthcare providers, enabling them to effectively leverage telemedicine technologies to deliver high-quality care in war zones.

## Conclusions and future considerations

Telemedicine holds immense potential to transform healthcare delivery in war zones, offering a lifeline to vulnerable populations and expanding access to vital medical services. However, realizing its full potential requires addressing the unique challenges posed by conflict and ensuring a comprehensive and integrated approach to healthcare delivery.

Moving forward, research and development efforts should focus on improving the accessibility and reliability of communication infrastructure in war zones, developing innovative telehealth technologies that are tailored to the specific needs of conflict-affected populations, building capacity and training for healthcare providers in the use of telemedicine, and advocating for policies that promote the ethical and responsible use of telemedicine in war zones.

By working collaboratively, governments, humanitarian organizations, and technology developers can harness the power of telemedicine to create a more equitable, accessible, and resilient healthcare system in war zones, ultimately saving lives and improving the health and wellbeing of vulnerable populations.
